# Fractional Exhaled Nitric Oxide in Teenagers and Adults with Atopic Dermatitis

**DOI:** 10.3390/arm90040033

**Published:** 2022-07-20

**Authors:** Sabina Galiniak, Marta Rachel

**Affiliations:** 1 Institute of Medical Sciences, Medical College, Rzeszów University, Warzywna 1a, 35-310 Rzeszów, Poland; rachel@popia.pl; 2 Department of Allergology and Cystic Fibrosis, State Hospital 2 in Rzeszów, Lwowska 60, 35-301 Rzeszów, Poland

**Keywords:** atopic dermatitis, exhaled nitric oxide, SCORAD, skin prick tests

## Abstract

**Highlights:**

- Fractional exhaled nitric oxide is significantly higher in patients with atopic dermatitis without asthma and allergic rhinitis.
- Fractional exhaled nitric oxide is positively correlated with the number of positive skin prick tests in patients with atopic dermatitis.
- Fractional exhaled nitric oxide is not associated with disease severity and the SCORAD index among patients with atopic dermatitis.

**Abstract:**

Fractional exhaled nitric oxide (FeNO) is a non-invasive biomarker of eosinophilic airway inflammation and therapeutic response to corticosteroid treatment of respiratory diseases. Atopic dermatitis (AD), one of the most common allergic conditions of the skin, is a factor influencing the increase of FeNO. The main aim of this study was to determine differences between levels of FeNO in patients with AD and healthy controls as measured by an electrochemical analyzer. In total, 54 teenagers and adults with AD were recruited and compared with 34 healthy volunteers. The measurements of FeNO were taken using the Hyp’Air FeNO in participants. FeNO was statistically significantly higher in patients with AD than in healthy controls (60.5 ± 35.1 vs. 14.8 ± 5.1 ppb, *p* < 0.001). We found a strong positive significant correlation between FeNO and the number of positive skin prick tests among AD patients (R = 0.754, *p* < 0.001). There was no correlation between FeNO and duration of disease as well as SCORAD index among patients. Moreover, we also found no FeNO difference between the mild and moderate forms of AD. The presence of AD and the increasing number of positive skin prick tests increase FeNO, so the results of this measurement should be interpreted with caution in patients with respiratory diseases suffering from AD.

## 1. Introduction

Atopic dermatitis (AD) is one of the most common allergic conditions of the skin with a wide spectrum of clinical presentations and combinations of symptoms. AD affects up to 20% of children and up to 3% of adults [1,2]. The pathogenesis of AD is multifactorial, and presumably originates from the interplay of genetic, immunologic, and environmental factors, infections causing dysfunctions of the skin barrier, and inflammation [3]. AD is associated with an increased risk of multiple comorbidities, including food allergy, asthma, allergic rhinitis, and mental health disorders [4,5]. AD is one of the factors influencing the level of fractional exhaled nitric oxide (FeNO) [6,7]. FeNO is used as a non-invasive marker for determining the degree of inflammation and efficiency of corticosteroid treatment. Elevated FeNO was observed in children and adults with asthma, allergic rhinitis, combined asthma, and allergic rhinitis as well as a chronic obstructive pulmonary disease [8,9,10]. Due to the growing number of reports on the influence of many factors on FeNO, FeNO is beginning to be considered an invaluable tool for the detection of asthma in patients with AD.

The primary aim of this study was to find out the differences between levels of FeNO in AD teenagers and adults compared to healthy controls. Moreover, we have made an attempt to estimate the factors influencing the increase of FeNO in participants with AD.

## 2. Material and Methods

### 2.1. Ethical Aspects

All participants gave written informed consent, and the study was approved by the Bioethical Commission of Rzeszów University (approval numbers 7/10/2018 and 22/02/2019). All procedures performed in studies involving human participants were in accordance with the ethical standards of the institutional and/or national research committee and with the 1964 Helsinki declaration and its later amendments or comparable ethical standards. 

### 2.2. Study Population 

**Figure 1:**
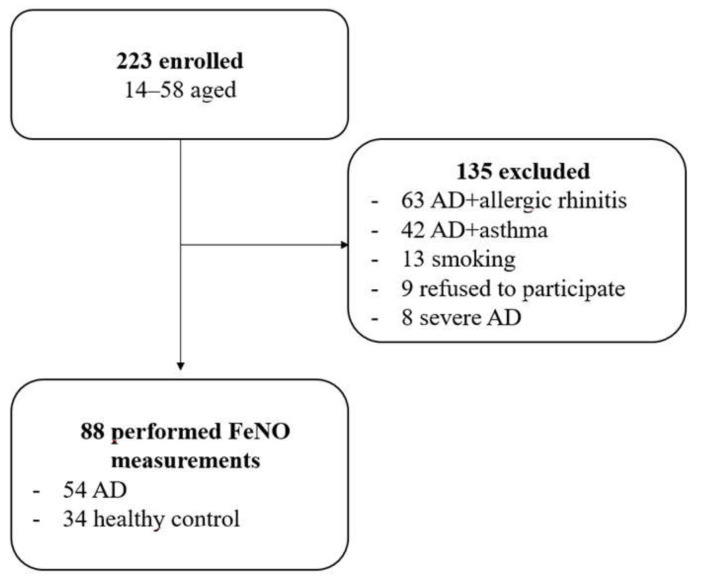
Flow chart of number of patients recruited and analyzed in the study.

A study was conducted among 189 patients aged 14–58 attending the Allergology Outpatient Department, Provincial Hospital No. 2 (Rzeszów, Poland) for treatment of AD from 1 April 2019 to 31 July 2019. Among 189 treated AD patients there were 63 patients with allergic rhinitis and 42 with asthma who had been excluded. Moreover, 22 patients with AD were excluded from the study due to smoking and refusal to participate in the study. Additionally, 8 patients were excluded due to severe AD (Figure 1).

At the same time, age- and sex-matched control subjects (*n* = 34) without respiratory diseases, allergy, AD, and atopy in clinical history were recruited. Participants in the control group had not taken any medication for 1 month prior to participation in the study. Healthy volunteers were recruited among the employees and patients of the other outpatient departments of Provincial Hospital No 2. Characteristics of the patients are shown in Table 1.

### 2.3. Inclusion Criteria

The inclusion criterion was AD as the main diagnosis, established on the basis of the Hanifin-Rajka criteria [11]. The patients in whom the primary diagnosis of AD raised any doubts were excluded from the study. Moreover, the SCORAD index was used to assess the extent and severity of AD. Patients were treated with topical medications such as emollients, calcineurin inhibitors (1% of pimecrolimus and tacrolimus), and antihistamines. Patients had not used topical steroids for 2 weeks prior to the study. Family history of atopy was defined as one or more of the following: physician-diagnosed asthma, suggestive history of allergic rhinitis/conjunctivitis, the physician diagnosed allergic eczema or any other confirmed IgE mediated allergies in at least one parent or sibling.

### 2.4. Exclusion Criteria

Exclusion criteria were as follows: asthma, allergic rhinitis, acute respiratory infections, the coexistence of other diseases, systemic corticosteroid treatment, lack of cooperation with devices used for respiratory tests, refusal to participate in the study, current smoking activity, and hospitalizations in the 1 month prior to screening. The diagnosis of asthma was made on the basis of GINA criteria, including respiratory symptoms in time and intensity as well as with the Polish version of the validated Asthma Control Test (ACT) questionnaire. The spirometry was used to confirm the reversibility of airway obstruction. ACT scores less than 20 are associated with uncontrolled asthma [12]. Patients were diagnosed with allergic rhinitis when they had one or more symptoms (nasal congestion, nose running, nasal itching, or sneezing) longer than 4 days per week and occurring after exposure to allergens [13].

Further exclusion criteria included: food allergies, severe AD, and other skin diseases such as seborrheic dermatitis, ichthyosis, psoriasis, scabies, contact eczema, cutaneous lymphomas, and other skin lesions associated with systemic diseases. Moreover, patients taking cyclosporin, methotrexate, and azathioprine were excluded.

### 2.5. Exhaled FeNO Measurements

The concentration of FeNO was measured using the Hyp’Air FeNO electrochemical device (MediSoft, Belgium) with a measuring range of 0–600 ppb in the case of bronchial measurement. The analyzer was calibrated and used according to the manufacturer’s instructions. Nitric oxide was measured three times for each patient according to the recommendations of the American Thoracic Society (ATS) [14]. Nitric oxide measurement is the exhalation of air through a disposable mouthpiece under a constant flow of 50 mL/sec for 6 s. Participants did not eat, drink, smoke, or exercise within 3 h prior to measurement. FeNO was always measured prior to spirometry to avoid any interference.

### 2.6. Other Tests

Spirometry was performed using a standard spirometry device (Lungtest 10,000 MES SJ, Krakow, Poland) according to recommendations of the ATS and European Respiratory Society [15]. The patients underwent blood sampling for blood cell count and basic biochemical analysis (C-reactive protein and total IgE in serum). Also, all patients had allergy tests with a panel of the most common aero-allergens (Skin prick tests—BASIC SET inhaled Allergopharma, Germany): *Dermatophagoides pteronyssinus*, *D. farina*, cat dander, dog dander, grass and tree pollens. The levels of specific IgE for *D. pteronyssinus* and *D. farinae* were measured with the ImmunoCAP ISAC kits.

### 2.7. Statistical Analysis

Data are given in the form of arithmetic mean values and standard deviations. Statistical significance of differences was evaluated using the Mann-Whitney U test. Spearman’s rank correlation coefficient analysis was employed to estimate the relationships between FeNO and other factors, assuming linear dependence. Statistical analysis of the data was performed using the STATISTICA software package (version 13.3, StatSoft Inc. 2017, Tulsa, OK, USA).

## 3. Results

Between April and July 2019, FeNO levels were measured successfully in 88 volunteers. Altogether, we enrolled 54 patients with AD (61.4%) and 34 healthy control subjects of comparable age and sex (Table 1). 

There were no differences in volunteers’ age, BMI, WBC, and spirometry results. We found significantly elevated levels of eosinophils and total IgE in patients with AD than in healthy controls (*p* < 0.001). Moreover, CRP was significantly increased in people with AD as compared to healthy participants (*p* < 0.05). Duration of atopic dermatitis, SCORAD index as well as the most common allergies and level of specific IgE among AD patients are presented in Table 1. Among the AD patients, there were 35 mild and 19 with moderate AD. There were no active smokers among the study participants, however, there were former smokers in both healthy subjects (11.8%) and patients with AD (13%) who declared not to have smoked for a minimum of 8 years.

**Figure 2:**
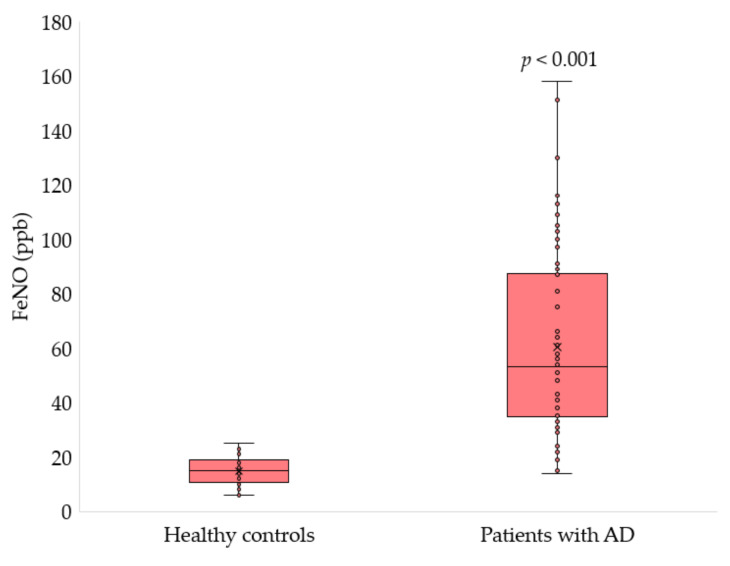
Comparison of FeNO in healthy and AD subjects, data are presented as mean ± SD, and differences between means were analysed using the Mann–Whitney test using Statistica software (version 13.3, StatSoft Inc. 2017, Tulsa, OK, USA).

Figure 2 presents the measured FeNO levels in healthy subjects and AD patients. FeNO was statistically significantly higher in patients with AD than in healthy controls (60.5 ± 35.1 vs. 14.8 ± 5.1 ppb, *p* < 0.001). The range of FeNO in AD participants was from 14 to 158 ppb, while in healthy people it was from 6 to 25 ppb.

We did not find a difference in FeNO between women and men with AD (65±35.8 vs. 56.7 ± 34.6 ppb, respectively, *p* = 0.52). Dependence between FeNO level and a patient’s general characteristics, clinical hematologic variables, and serum biochemical parameters were estimated by using Spearman correlation. A weak positive correlation was estimated between FeNO and patients’ age (R = 0.34, *p* = 0.011).

**Figure 3:**
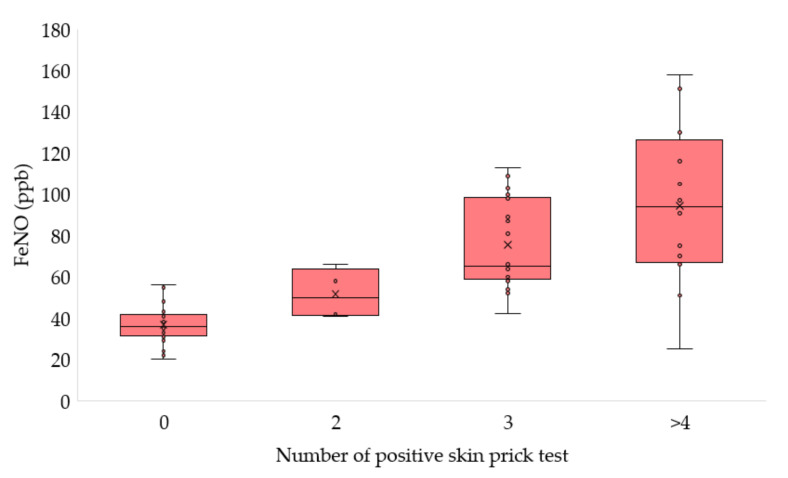
Comparison of FeNO in AD patients depending on the number of positive skin prick tests, data are presented as mean ± SD.

Additionally, we found a strong positive significant correlation between FeNO and the number of positive skin prick tests among AD patients (R = 0.754, *p* < 0.001, Figure 3). 

Nevertheless, we did not notice any additional associations between FeNO and BMI, hematologic variables, biochemical parameters, and spirometry results. Surprisingly, there were also no correlation between FeNO and duration of disease as well as SCORAD index among patients (R = 0.008, *p* = 0.95 and R = 0.114, *p* = 0.43, respectively). Moreover, we also found no FeNO difference between the mild and moderate forms of AD (60.5 ± 33.1 vs. 60.4 ± 39.3 ppb, respectively, *p* = 0.785).

## 4. Discussion

To our knowledge, this is the first report showing the distribution of FeNO not only in the teenage population but also in adults with AD. The distribution of FeNO was wide between AD patients and ranged from 14 to 158 ppb which was statistically higher than the FeNO level in the controls. The wide dispersion of FeNO results among AD patients was also noticed in the study of Zinelli et al. [16]. 

Similar to our results, FeNO was significantly higher in atopic children than healthy controls from Spain (19 vs. 7.9 ppb, *p* < 0.001) [17] as well as China (20.9 ± 1.9 vs. 13.2 ± 1.9 ppb, *p* = 0.074) [18]. Moreover, this observation in children and young adults was confirmed by van Asch et al. [6] and Welsh et al. [19]. The sex of the study participants is one of the parameters that may influence the FeNO level [7]. Nevertheless, we did not notice a FeNO difference between men and women. No differences in FeNO between male and female participants were also documented in the study of Hon et al. [20].

The effect of age on FeNO is inconclusive. Some studies indicate that age affects FeNO, and others that age is an independent factor [8,9,21,22]. However, the influence of age on FeNO might be associated with geometrical factors such as the total area of airway mucosa which produces NO [23].

The exact basis of the elevated FeNO levels in people with atopy is currently unclear, however, some scientists have suggested that it is not atopy as such that causes higher FeNO levels, but a combination of an allergic state and exposure to an appropriate allergen that leads to allergic inflammation [24]. In our study, we noticed a trend that FeNO increased with an increasing number of positive skin tests for aeroallergens. Likewise, FeNO was positively correlated with the number of positive skin prick tests in a group of participants aged 18 [25]. FeNO levels varied according to the profile of atopy, as determined by positive skin prick test results for various classes of aeroallergens in a study by Jang et al. [26]. Moreover, reported pollen/furry pet allergy was positively associated with an increase in FeNO among Chinese children in a study by Zhao et al. [27].

Subsequently, we did not find any differences in FeNO between mild and moderate AD. In a study of Zinelli, FeNO was significantly higher in children with severe AD compared with those with less severe AD (mild AD 50.06 ± 34, moderate AD 56.06 ± 21.5, severe AD 88.8 ± 73.8 ppb, *p* = 0.012) which confirms our observation [16]. Nevertheless, no correlation between FeNO and AD severity was found in Chinese children with AD. Moreover, similarly to our results, FeNO levels were not correlated with SCORAD index in these patients with AD [20].

It can be speculated that high levels of NO in patients may indicate a mild degree of lung inflammation that was asymptomatic at the time of the study and undetectable at that time by standard lung function tests [28]. In addition, studies show that in AD patients, inducible nitric oxide synthase is expressed in the skin, allowing NO to enter the lungs via the circulation [29]. It is known that serum NO level was significantly higher in AD patients than in controls [30,31]. Akdeniz et al. did not notice any significant difference in serum NO between mild and moderate AD. However, a significant difference was found between mild and severe groups, and between moderate and severe groups [30]. A positive correlation between serum nitrate level and grades of skin scores was observed in children aged 0.4–8 years [31].

In AD patients, NO production is associated with itch while inhibition of nitric oxide synthase suppresses itch [32].

Interesting findings were pointed out in our study, however, several limitations of the study have to be mentioned. Firstly, this study analyzed a small sample size. Subsequently, we analyzed patients from the age of 14, not younger. Moreover, the study was single-center. However, this study also has strengths. We believe that the strength of the study is its duration. Patients were equally exposed to allergens because the effects of pollen, animal allergens, and house dust were well matched. Nevertheless, the measurements of FeNO and nNO were performed at times of high pollen concentration (from April to July), which could have influenced the measurement results in susceptible individuals.

## 5. Conclusions

In conclusion, FeNO measurements are not a reliable tool to assess inflammation in patients with respiratory disease and mild or moderate AD. AD and the number of positive skin prick tests increase FeNO, so the results of this measurement should be interpreted with caution.

## Figures and Tables

**Table 1:** Demographic and clinical characteristics of the studied groups.

		Healthy Controls	Atopic Dermatitis	*p*
*n*		34	54	
Sex (female)	*n* (%)	22 (64.7)	28 (51.9)	
Age (years)	Mean ± SD	27.88 ± 12.3	26.61 ± 12.2	0.503
	range	14–53	14–55	
Duration of AD (years)	Mean ± SD	0	10.51 ± 7.6	
	range	0	1–30	
SCORAD index	Mean ± SD	-	21.3 ± 11.7	
	range	-	7–46	
Family history of atopy, *n* (%)		-	7 (13)	
BMI (kg/m^2^)	Mean ± SD	23.61 ± 2.8	23.6 ± 3.4	0.827
	range	18.25–28.4	17.57–30.92	
WBC (×10^3^/µl)	Mean ± SD	7 ± 1.7	6.74 ± 1.3	0.693
	range	4.58–11	4.23–9.54	
%EOS	Mean ± SD	1.53 ± 1.2	4.9 ± 4.2	0.0001
	range	0.3–4.5	0.3–15.5	
EOS (×10^3^/µl)	Mean ± SD	0.12 ± 0.1	0.38 ± 0.3	0.0001
	range	0.02–0.49	0.02–0.96	
CRP (mg/l) (reference range: 0–5 mg/l)	Mean ± SD	2.15 ± 1.6	2.92 ± 1.4	0.013
	range	0.3–6.4	0.2–6.6	
total IgE (kU/l) (reference range: adults and children < 10 years: < 25 kU/l—negative, 25–100 kU/l—doubtful, > 100 kU/l—positive)	Mean ± SD	21.51 ± 17.4	269.07 ± 59.7	0.0001
	range	1.4–61.2	160–374	
FVC (%)	Mean ± SD	111.26 ± 14.7	106.52 ± 12.4	0.181
	range	87–146	85–144	
FEV_1_ (%)	Mean ± SD	108.15 ± 13.9	102.83 ± 11.4	0.105
	range	81–145	82–128	
FEV_1_/FVC (%)	Mean ± SD	98.79 ± 6.2	97.11 ± 6.5	0.235
	range	85–115	83–118	
Smoking status	never smokers, *n* (%)	30 (88.2)	47 (87)	
	former smokers, *n* (%)	4 (11.8)	7 (13)	
Allergens	*Dermatophagoides pteronyssinus* and/or *D. farinae*, *n* (%)	0	34 (63)	
	cat and/or dog dander, *n* (%)	0	18 (33)	
	grass pollen, *n* (%)	0	22 (40)	
	tree pollen, *n* (%)	0	18 (33)	
Specific IgE level (kU/l)— *D. pteronyssinus*	Mean ± SD	0	27.67 ± 40.3	
	range	0	0–100	
Specific IgE level (kU/l)—*D. farinae*	Mean ± SD	0	29.61 ± 41.9	
	range	0	0–100	

Data are shown ± SD as mean as well as range or number of patients and percent, differences between means were analysed using the Mann-Whitney test using Statistica software (version 13.3, StatSoft Inc. 2017, Tulsa, OK, USA).

## Data Availability

Data supporting the results of this study shall, upon appropriate request, be available from the corresponding author.
